# The Yak genome database: an integrative database for studying yak biology and high-altitude adaption

**DOI:** 10.1186/1471-2164-13-600

**Published:** 2012-11-07

**Authors:** Quanjun Hu, Tao Ma, Kun Wang, Ting Xu, Jianquan Liu, Qiang Qiu

**Affiliations:** 1 State Key Laboratory of Grassland Agro-ecosystem, College of Life Science, Lanzhou University, Lanzhou, China

**Keywords:** Yak genome, Genome database, High-altitude adaption, Bovine genomics

## Abstract

**Background:**

The yak (*Bos grunniens*) is a long-haired bovine that lives at high altitudes and is an important source of milk, meat, fiber and fuel. The recent sequencing, assembly and annotation of its genome are expected to further our understanding of the means by which it has adapted to life at high altitudes and its ecologically important traits.

**Description:**

The Yak Genome Database (YGD) is an internet-based resource that provides access to genomic sequence data and predicted functional information concerning the genes and proteins of *Bos grunniens*. The curated data stored in the YGD includes genome sequences, predicted genes and associated annotations, non-coding RNA sequences, transposable elements, single nucleotide variants, and three-way whole-genome alignments between human, cattle and yak. YGD offers useful searching and data mining tools, including the ability to search for genes by name or using function keywords as well as GBrowse genome browsers and/or BLAST servers, which can be used to visualize genome regions and identify similar sequences. Sequence data from the YGD can also be downloaded to perform local searches.

**Conclusions:**

A new yak genome database (YGD) has been developed to facilitate studies on high-altitude adaption and bovine genomics. The database will be continuously updated to incorporate new information such as transcriptome data and population resequencing data. The YGD can be accessed at http://me.lzu.edu.cn/yak.

## Background

Yaks (*Bos grunniens*) inhabit the Qinghai-Tibetan Plateau and adjacent highlands, where they are an important source of basic living resources such as meat, milk, dung (which is used as fuel) for Tibetans and nomadic pastoralists [1]. Genome-wide comparisons between the yak and closely related cross-fertile taurine cattle (*B. taurus*) [2], which suffers from severe pulmonary hypertension when reared in the yak's natural habitat [3-5], can be used to study the processes of natural selection that drove leading to high-altitude adaptation [6].

## Construction and content

The YGD currently provides access to yak genome assembly version 1.1, a de novo genome sequence assembly prepared using the second generation sequencing technologies by State Key Laboratory of Grassland Agro-Ecosystem, College of Life Science, Lanzhou University, Lanzhou, China and BGI-Shenzhen, China. The sequence files are normally maintained by the former. The 65X genome assembly has a scaffold N50 of 1.4Mb with a total size of 2,657 Mb. It is thus similar in size to the 2,649 Mb cattle genome (UMD 3.1).

### Search

A search function was developed to facilitate the identification of genes based on their predicted annotations. Gene datasets were obtained using a variety of strategies, including RNA-seq, homology and *ab initio* gene prediction. For homology prediction, pseudo-genes were detected and dropped, gene models don’t have synteny support but have higher quality score and less frame shift were retained. For *ab initio* prediction, only gene models that have a minimum coverage of 30% in SwissProt/TrEMBL were retained. A consensus non-redundant dataset containing 22,282 protein-coding genes was built by merging different gene datasets using GLEAN (http://sourceforge.net/projects/glean-gene). Of these, 15716 (70.53%) have RNA-seq support, 21474(96.37%) of the predicted yak genes have a homologue (TreeFam) either in human (n=19,894; 89.28%), cattle (n=20,346; 91.31%) and dog (n=19,455; 87.31%) with 18,040 being homologous among all species examined, and 170 (0.76%) being ‘unique’ to yak. And 8923(40.05%) genes have single orthologs in bovine and human. This combined gene dataset was used as reference and has been integrated into the YGD. Predicted noncoding genes, including 481 miRNAs, 562 rRNAs and 499 tRNAs were also integrated into the database. The incorporated genes were annotated using a variety of methods including Swiss-Prot annotation, TrEMBL annotation, KEGG annotation, InterPro domain annotation and Gene Ontology annotation. Swiss-Prot and TrEMBL annotations for the predicted yak proteins were generated by performing BLASTP (e-value ≤10^-5^) searches for each one against the Swiss-Prot and TrEMBL databases. The corresponding yak genes were then mapped to KEGG pathway maps based on best BLASTP (e-value ≤10^-5^) hit. InterProScan was used to annotate motifs and domains in yak genes by comparing them to hits from the public Pfam, PRINTS, PROSITE, ProDom and SMART using the HMMPfam, FPrintScan, ScanRegExp, ProfileScan, BlastProDom and HMMsmart with the following parameters -format raw -goterms -iprlookup. Gene Ontology information was then extracted from the InterProScan results with in-house Perl scripts. These annotations will be refreshed when gene models are updated.

### Tools

#### Generic genome browser

We used the Generic Genome Browser (GBrowse) developed as part of the Generic Model Organism Database project (GMOD; http://gmod.org/wiki/GMOD) to visualize the genome of the yak [7]. In addition, predicted genes, single nucleotide variants (SNVs), multiple types of RNA sets and repeats contained within the YGD can be visualized using Gbrowse. To identify SNVs, high quality reads (i.e. reads with an average quality score above 30) from short insert size libraries were re-aligned to the assembly using SOAP (http://soap.genomics.org.cn/). The probabilities of each possible genotype at every position on the reference genome were calculated, and a statistical model based on Bayesian theory and the Illumina quality system was used to call SNVs. The allelic sequence with the highest probability was used as the reference sequence and heterozygosity was calculated based on other high probability alleles. Repeat sequences were identified by two different methods. First, we identified known TEs using RepeatMasker (http://www.repeatmasker.org) and the Repbase TE library (http://www.girinst.org/repbase), and then used the RepeatProteinMask program to identify TEs by aligning the genome sequence to a self-generated curated TE protein database. Second, we constructed a de novo yak repeat library using RepeatModeler, which yielded consensus sequences and classification information for each repeat family. The RepeatMasker program was then applied to these genome sequences, using the RepeatModeler consensus sequences as the library. GBrowse makes it possible for users to easily browse any region of interest in the yak genome. A variety of track features can be accessed depending on the region's position on the scaffold, including protein-coding genes, noncoding genes, GC content and repetitive sequences.

#### BLAST

BLAST is one of the most useful tools for searching the YGD, offering users the ability to search against scaffolds and genes in Yak 1.1. On the results page for a BLAST search of YGD, each hit is linked to the GBrowse view of the sequence.

#### Generic synteny browser

A three-way genome comparison between human, cattle and yak was generated using Mercator and MAVID [8], and can be viewed using the Generic Synteny Browser (Gbrowse-syn), which based on the same software framework as GBrowse. The Gbrowse-syn can be used to compare co-linear regions of multiple genomes using the familiar GBrowse-style web page and can also display gene symbols and functional information.

#### Downloads

Data from the YGD can be downloaded to perform local searches against the whole genome, specific gene sequences and mitochondrial genomes.

## Utility and Discussion

**Figure 1:**
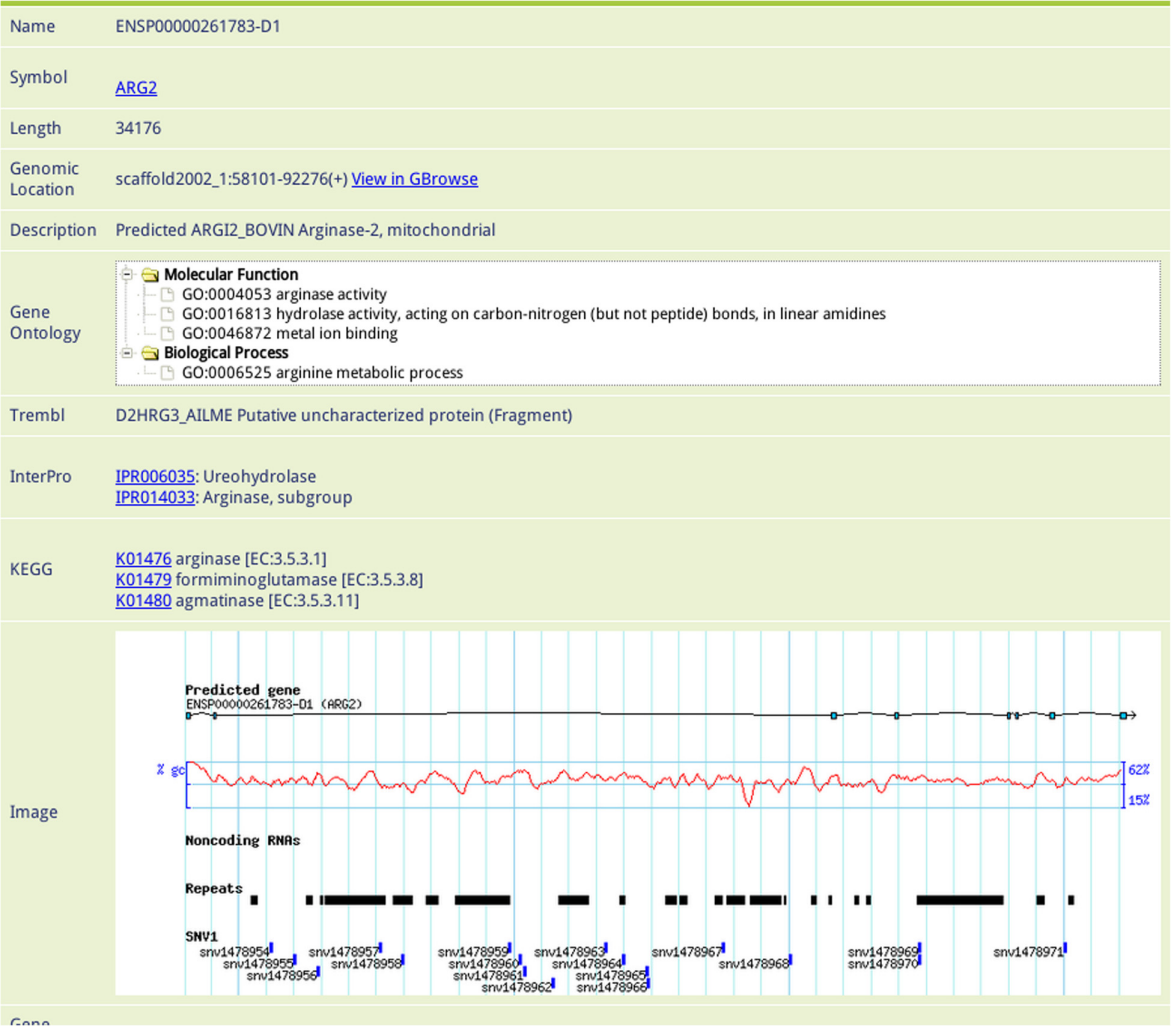
**Gene information page. **Gene information page containing all available information related to the gene in question. The gene description is extracted from its Swiss-Prot annotation. Gene Ontology data are grouped by domain. Clicking on the gene's ID (e.g. IPR006035, K01476, etc.) will cause a new window to appear providing information about the ID. The image provides an overview of the gene's structure and related features.

**Figure 2:**
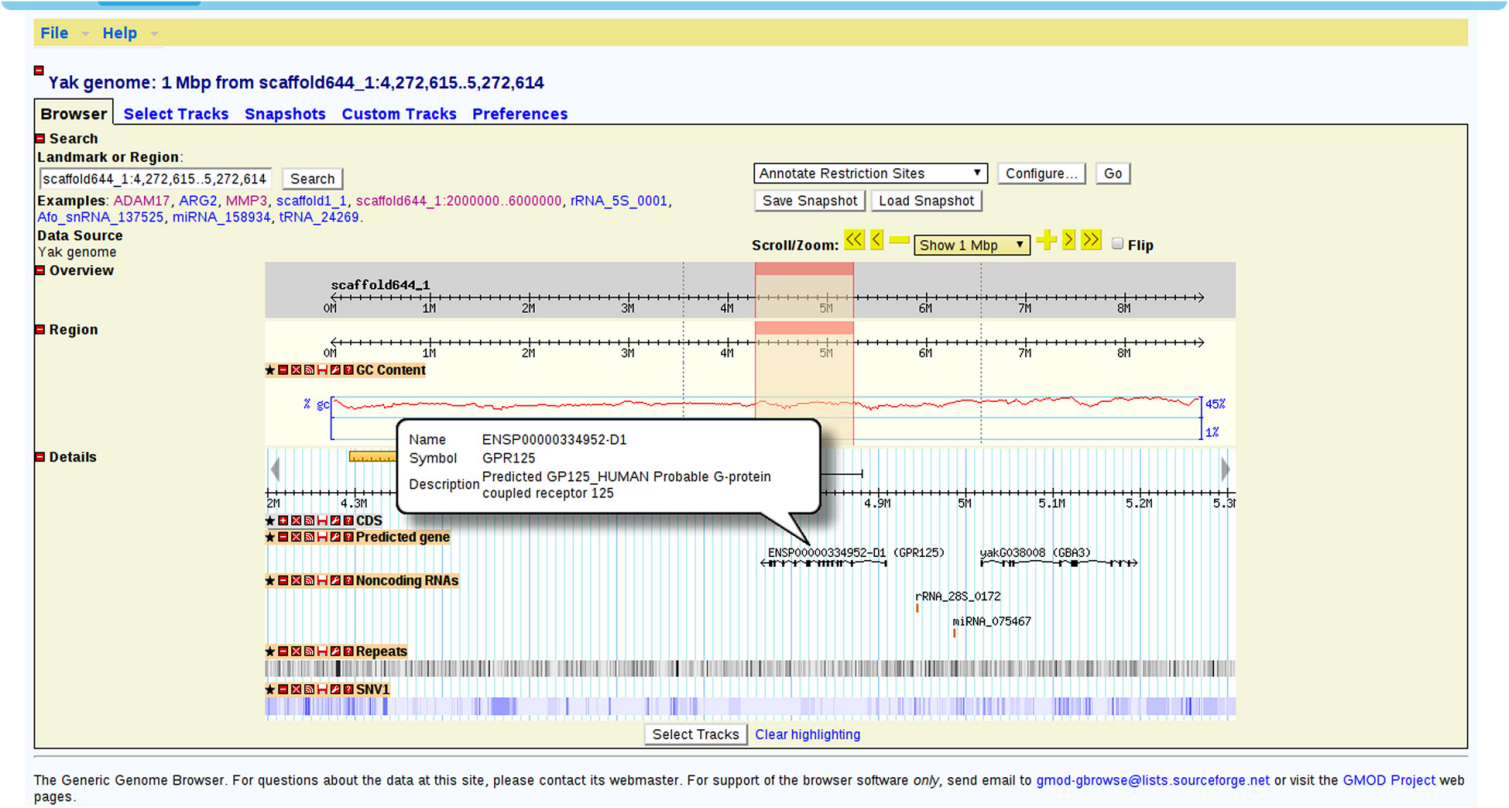
**Visualization of genome annotation using GBrowse. **There are four main tracks in GBrowse: predicted genes, noncoding RNAs, repeats and SNVs. When the user holds the cursor over a predicted gene, a popup balloon will appear showing gene information. Repeats and SNV1 are displayed in a density graph; more detail can be obtained by zooming in.

**Figure 3:**
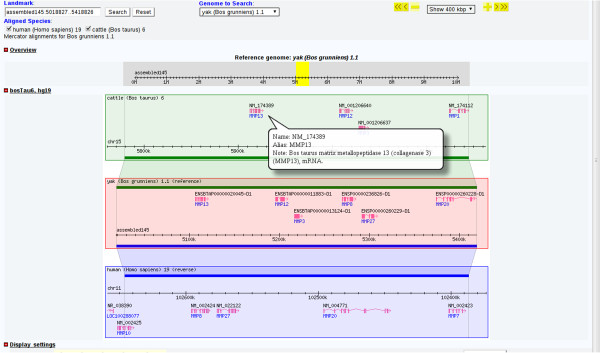
**Genome alignment between human, cattle and yak. **Alignments are shown using GBrowse syn. The gene id is shown at the top of the gene and the gene symbols on the bottom. If the user hovers over a specific gene, a popup balloon will appear to provide more information on it.

**Figure 4:**
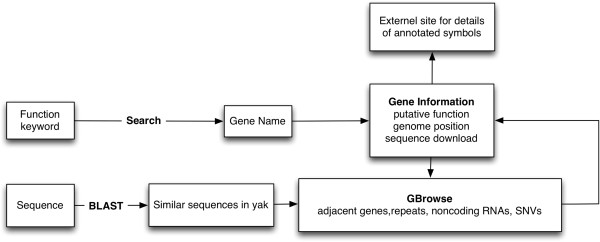
**A schematic overview of the information flow in YGD. **The query information can be input as a keyword or a sequence, then using Search or BLAST to retrieve related information in database and view details in gene information page or GBrowse. In addition, user can click annotated symbols to view details about the symbol in external website.

In the search page, users can find specific genes by searching using keywords, such as “transferase” or “transporter”. Information on individual genes can then be accessed by clicking gene's name or annotated identifier to see its description at appropriate external website. In the gene information page, the user can view the gene's annotated description, jump to GBrowse or download gene data. This page also provides a quick overview of the gene's structure and related information (Figure 1). GBrowse allows users to view different elements on different tracks, including predicted genes, non-coding RNAs, repeats and SNVs. If the user zooms out past a certain range, summary mode will be turned on, which displays a density graph for certain features. If the user then hovers over a predicted gene, a popup balloon will appear showing gene symbols and providing functional information. Alternatively, by clicking on a specific gene, the user can jump to the corresponding gene information page to obtain more information about it; once done, the user can return to the zoomed out view by clicking on the “Tools” section of the navigation bar at the top of the window (Figure 2). The Generic Synteny Browser enables the user to visualize data on multiple sequence alignments between human, cattle and yak genomes. As with GBrowse, a popup balloon will be shown if the user hovers over a specific gene (Figure 3). A schematic overview of the information flow in the YGD is shown in Figure 4.

The Bovine Genome Database http://genomes.arc.georgetown.edu/drupal/bovine was established to support the efforts of the Bovine Genome Sequencing and Analysis Consortium by creating a model organism database that integrates bovine genomics data with structural and functional annotations of genes and the genome. Conversely, the YGD was created as a tool to share yak genome resources and facilitate comparative genomic studies between the yak, humans, and cattle, thereby facilitating the study of high-altitude adaptation in animals. New data will be promptly incorporated into the YGD as they become available. A number of research projects focusing on the yak are currently in progress, including the yak transcriptome project (which focuses on studying gene expression profiles in different yak tissues) and the resequencing project. The experimental data from these studies and the results of the corresponding analyses will be integrated into the database.

## Conclusions

A yak genome database (YGD) has been developed to facilitate studies of high-altitude adaptation and bovine genomics. The YGD provides new data on the yak genome that is not available in the existing genome databases, along with data-mining tools for studying high-altitude adaptation and bovine biology. The scope and applications of the YGD will be improved and expanded continuously in future and new datasets will be incorporated to help scientists and breeders to fully and efficiently exploit bovine genome-wide datasets. The YGD will be a valuable resource for scientists studying comparative genomics, molecular biology and extreme environment adaptation.

## Availability and requirements

**Database name:** YGD

**Database homepage:**http://me.lzu.edu.cn/yak

**Browser requirement:** JavaScript should be enabled; we recommend the use of the Chrome or Firefox web browsers for an optimal experience.

The data in the YGD are freely available for academic use. For all other uses, please contact Qiang Qiu (qiuq04@gmail.com).

## Abbreviations

YGD: Yak Genome Database; GBrowse: Generic Genome Browser; GBrowse syn: Generic Synteny Browser; SNVs: Single nucleotide variants.

## Competing interests

The authors declare that they have no competing interests.

## Authors' contributions

QQ, JL and QH conceived the study. QH processed the data and developed the database. QH prepared the manuscript; QQ and JL proofread it and provided critical feedback. KW tested the web application and tools and provided feedback. TX maintained the database. QQ and TM prepared the basic datasets. All authors read and approved the final manuscript.
